# Oculo-dento-digital dysplasia: a systematic analysis of published dental literature

**DOI:** 10.1038/s41405-023-00139-7

**Published:** 2023-03-29

**Authors:** Karshma Devi Hindu, Fahad Umer

**Affiliations:** Aga Khan University Hospital, Stadium Road, Karachi, 74800 Pakistan

**Keywords:** Endodontics, Caries risk assessment

## Abstract

**Introduction:**

Oculo-dento-digital dysplasia (ODDD, OMIM# 164200) is a rare genetic disorder caused by mutation in Gap junction alpha gene that encodes connexin 43 (Cx43) protein. In this paper, the case of a 16-year-old boy is reported who presented with the complaint of toothache. Examination revealed unusual facial features, i.e., long narrow nose, hypertelorism, prominent epicanthal folds along with syndactyly and camptodactyly. We have also compiled available dental literature on ODDD that will help clinicians in early diagnosis and management of this condition.

**Materials and methods:**

A literature search was performed in PubMed NLM, EBSCO Dentistry & Oral Sciences Source, and EBSCO CINAHL Plus.

**Results:**

A total of 309 articles were identified in the literature search. Only 17 articles were included based on the predetermined inclusion and exclusion criteria in the review synthesis. The included articles were case reports (*n* = 15), a case report and review (*n* = 1), and an original article (*n* = 1). Enamel hypoplasia, hypomineralization, microdontia, pulp stones, curved roots, and taurodontism were common dental findings in ODDD.

**Conclusions:**

After establishing definitive diagnosis, a multidisciplinary team should work in cohesion to improve the quality of life of patients. Immediate treatment should be focused on the correction of current oral condition and symptomatic treatment. In the long term, attention should be diverted to prevent tooth wear and maintaining the occlusal vertical dimension to establish adequate function.

## Introduction

Oculo-dento-digital dysplasia (ODDD, OMIM# 164200) is a rare congenital genetic disorder characterized by craniofacial, ocular, dental, and digital abnormalities [1]. It was initially recognized by Lohman in 1920 [2]. It is primarily an autosomal dominant disorder but, in a few cases, recessive forms of the disease have been identified [3]. ODDD is caused by a missense mutation in gap junction alpha 1 (GJA1) gene on chromosome 6q22.31 [3]. This gene encodes for connexin 43 (Cx43), a transmembrane protein [3]. In 2003, ODDD became known as the first human disease to be linked to germline Cx43 gene (GJA1) mutations [4]. Cx43 is one of the 20 members of the human connexin protein family [3]. It has been diagnosed in fewer than 300 people worldwide with an incidence of around 1 in 10 million [2]. ODDD has high penetrance and its phenotypic expression is variable [5, 6], ODDD has typical features (Table 1) of syndactyly [3], digital camptodactyly [7], ophthalmic [8], nasal [3], and dental abnormalities [9].

**Table 1:** Clinical features of the ODDD syndrome.

The hallmark features of the syndrome
• Syndactyly of the fourth and fifth fingers in both hands [3]. • Digits camptodactyly [7]. • Microphthalmia, microcornea, hypo/hypertelorism with short palpebral fissures [8]. • A depressed nose bridge, hypoplastic and anteverted nares [3]. • Hypoplastic enamel (40%) with caries, microdontia (21%), missing teeth (7%), amelogenesis imperfecta (2%), pulp stones (2%), and delayed tooth development (2%) [9].

Other than these mentioned features, ODDD also shows neurological [10–12], and cardiological involvement [13]. Some features of ODDD are evident at birth, while others may appear with increasing age. Despite undergoing medical treatment for eyesight, and multiple surgeries for syndactyly and camptodactyly in hands in childhood, the case presented here was first diagnosed in our dental practice. This shows that rare syndromes like ODDD can remain either undiagnosed or misdiagnosed. Therefore, this review aims to summarize the available dental literature on ODDD as it will enable better management of associated diseases to improve the quality of life of the patient.

## Case report

**Fig. 1:**
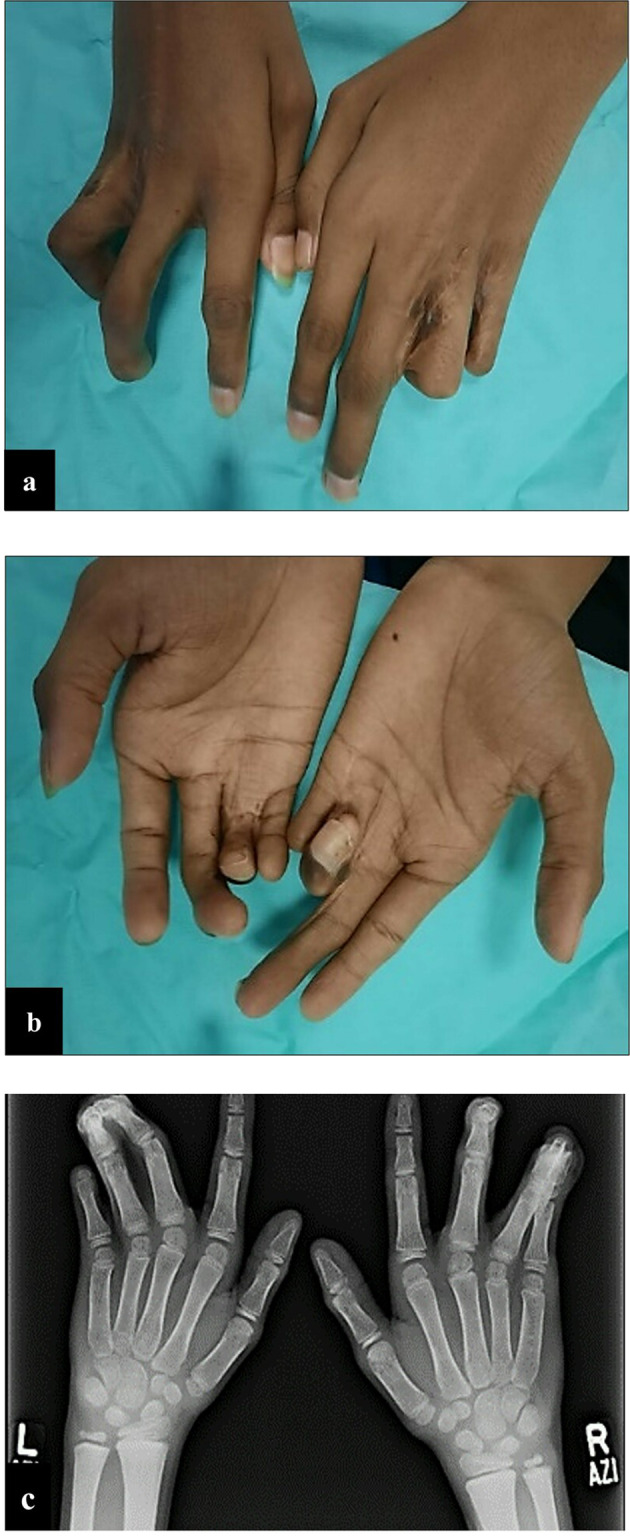
Hand photograph and radiograph illustrating webbed fingers. **a**–**c** Syndactyly of fourth-fifth fingers of right hand and third-fourth fingers of left hand with camptodactyly.

**Fig. 2:**
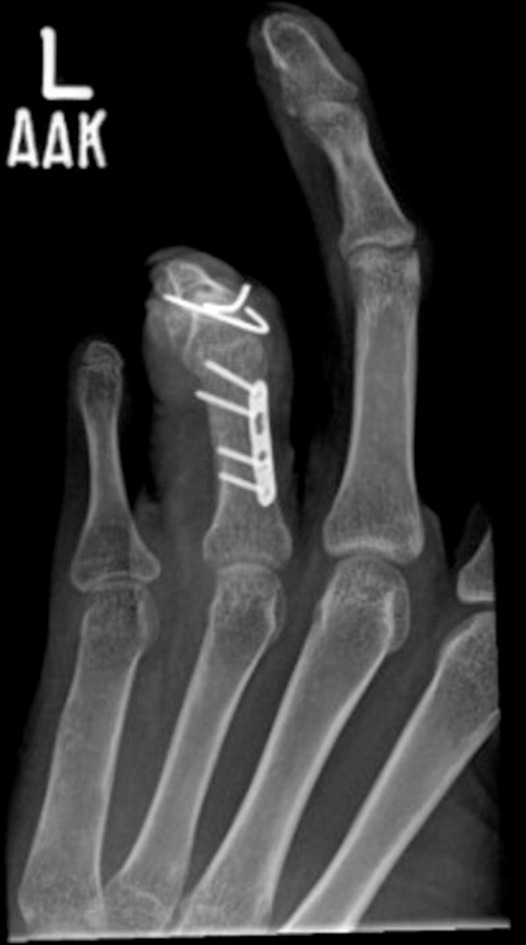
Post-operative hand radiograph. Post-operative x-ray *showing* separation of third and fourth fingers of left hand.

**Fig. 3:**
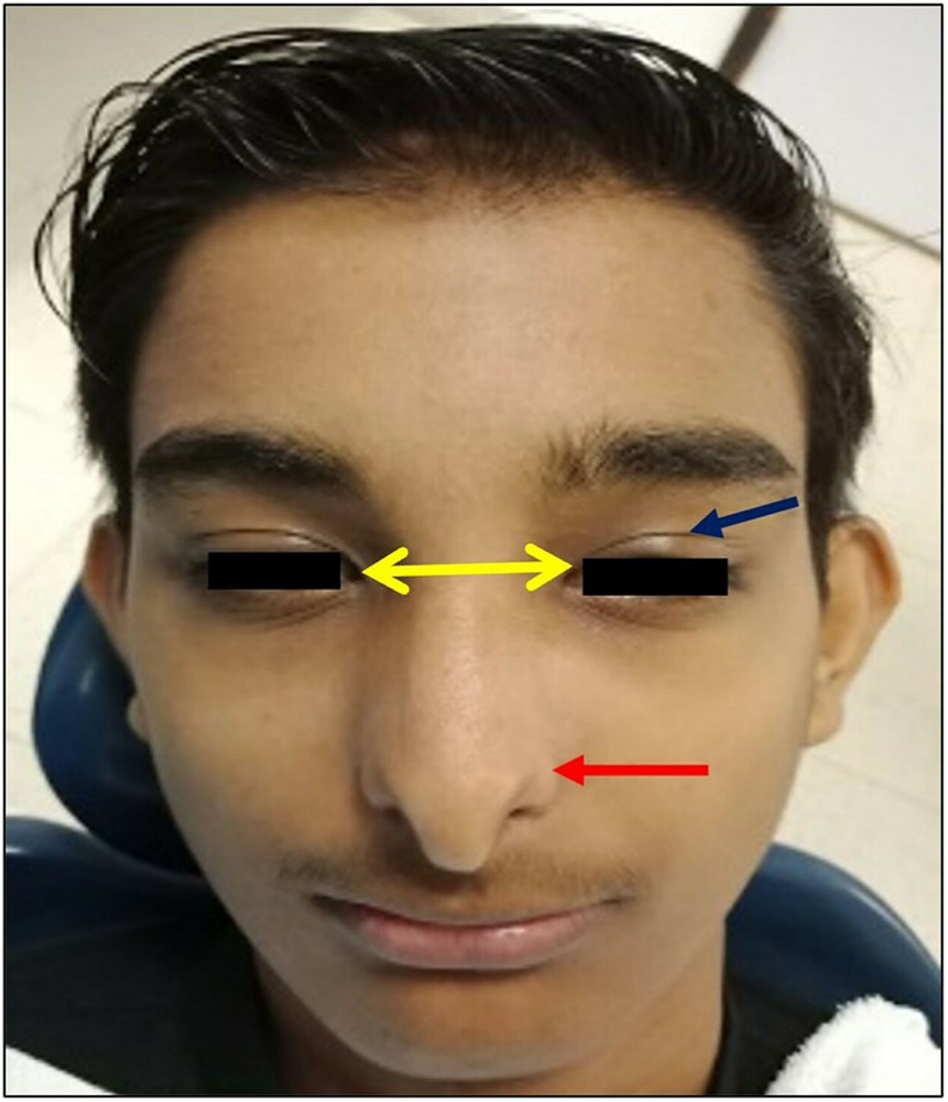
Extra-oral frontal photograph. Arrows showing prominent *epicanthal* folds (blue), Hypertelorism (yellow), Hypoplastic and anteverted nares (red).

A 16-year-old male patient visited the dental clinic in February 2020 with complaints of pain in the lower right posterior tooth and sensitivity to cold in all posterior teeth. On physical examination, the patient had syndactyly of fourth-fifth fingers of the right hand and third-fourth fingers of the left hand with camptodactyly and webbing (Fig. 1). Multiple surgeries were carried out from birth to 12 years of age, but digital abnormalities were still present. Another corrective surgery was done in 2021 which resulted in a successful resolution of syndactyly of left-hand digits (Fig. 2). On extra-oral examination patient’s nose was thin, elongated with hypoplastic alae nasi, anteverted nares and ocular findings included prominent epicanthic folds and hypertelorism (Fig. 3).

**Fig. 4:**
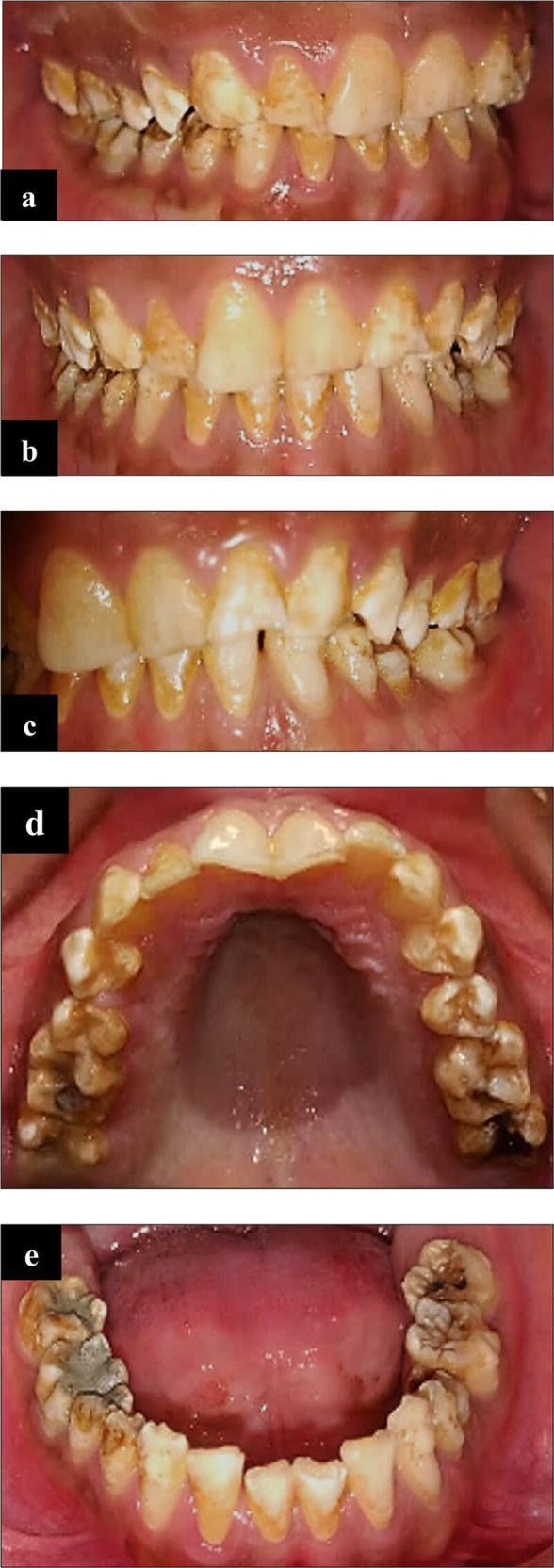
Intra-oral clinical photographs. **a** Right lateral view. **b** Frontal view. **c** Left lateral view. **d** Maxillary occlusal view. **e** Mandibular occlusal view showing generalized *staining*, hypoplastic enamel (most obvious on posterior teeth) and deep fissure and multiple carious teeth. Tooth #46 root canal treated and 47 was filled.

Upon intra-oral examination, findings were generalized staining, hypoplastic, hypomineralized enamel with pitting (most obvious on posterior teeth), multiple carious teeth: # 16, 17, 26, 27, 36, 37, 45, 46, 47, deep fissures in tooth # 25, 34, 35 (Fig. 4). Tooth # 46 was non-vital on electric pulp testing (EPT) and cold test and tender to percussion.

**Fig. 5:**
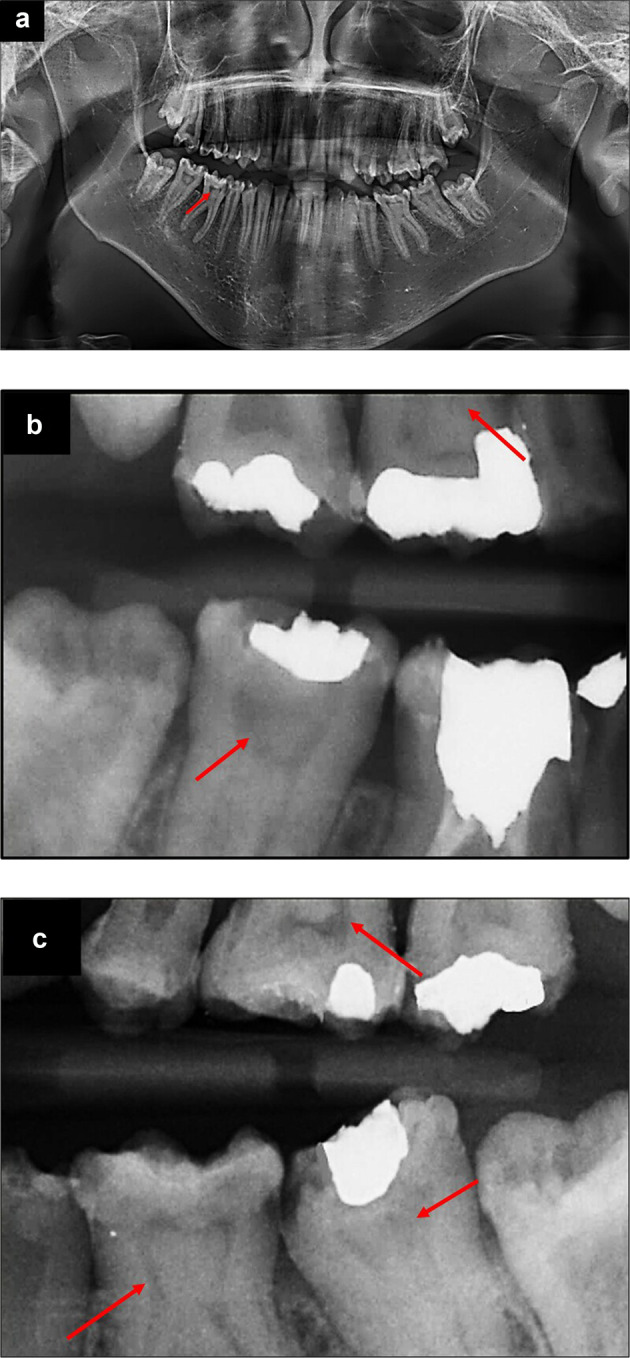
Dental radiography: Orthopantomogram and bitewing radiographs. Orthopantomogram (**a**) *showing* taurodontism in all upper, lower second and third molars with curved roots in lower first molars. Right (**b**) and left (**c**) bitewings showing pulp stones (red arrows) in all first molars and lower right, left second molars.

On panoramic (A) and right (B) and left (C) bitewing radiographs, all upper and lower second and third molars had taurodontism and pulp stones (red arrows) along with curved roots (Fig. 5).

### Management and follow-up

**Fig. 6:**
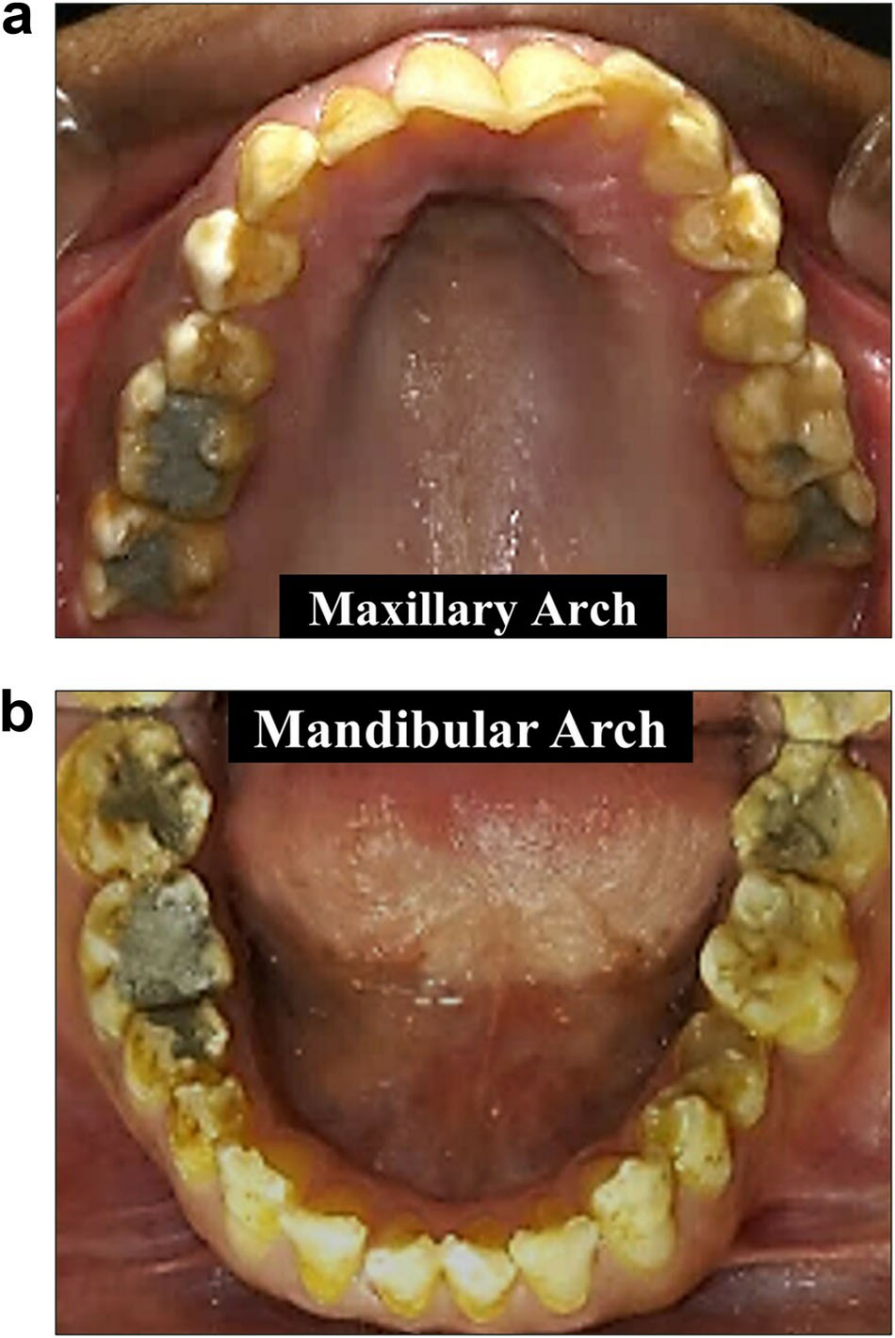
Post-operative occlusal photographs. **a**, **b** Showing maxillary and mandibular teeth after completion of dental treatment.

**Fig. 7:**
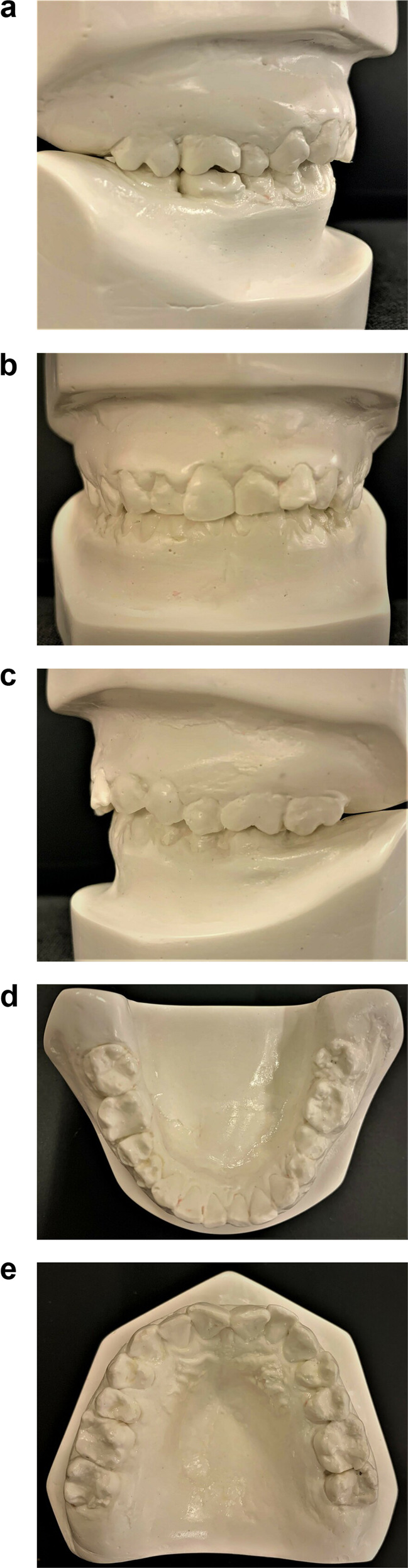
Diagnostic cast. **a**–**e** Diagnostic *cast* to monitor tooth wear on follow-up visits.

Orthograde endodontic treatment was initiated in tooth # 46, after copious sodium hypochlorite irrigation and intracanal medicament, temporary restoration was placed. After 1 week, endodontic treatment of tooth # 46 was completed followed by permanent restoration. Restoration of all carious teeth # 16, 17, 26, 27, 36, 37, 45, 46, 47 was done with amalgam filling material (Fig. 6). Deep fissures in tooth #25, 34, 35 were sealed with light cure resin composite (Fig. 6). Dietary modification and oral hygiene instruction were reinforced. Impressions were obtained for diagnostic casts to monitor tooth wear on follow-up visits (Fig. 7). A 3-monthly follow-up was scheduled to evaluate the further enamel loss of the unrestored dentition and check the restored teeth status. The patient was asymptomatic and tooth sensitivity had diminished. No further deterioration of enamel was apparent.

## Review questions

We decide to systematically look into these cases as no such review has been published till now. For this review the authors (KDVH & FUMR) compiled the available dental literature on ODDD patients based on the following questions:

1. What are the diagnostic measures, i.e., clinical features or gene analysis?
2. What are the dental manifestations commonly seen in patients with ODDD?
3. What are the management options for dental abnormalities?

## Materials and methods

### Search strategy

The authors (KDVH & FUMR) conducted a pilot search based on various combinations of key search terms. The final search strategy was formulated based on this pilot search. A comprehensive online literature search was performed (in June 2021) in three major health sciences databases, PubMed NLM, EBSCO Dentistry & Oral Sciences Source, EBSCO CINAHL Plus, and a hand search was also done in collaboration with a medical information specialist (Librarian, Aga Khan University Hospital, Pakistan).

### Search terms

(“Oculodentodigital Dysplasia” [Supplementary Concept] OR Oculodentodigital dysplasia OR Oculo-dento-digital dysplasia OR Oculodentodigital syndrome OR Oculo-dento-digital syndrome OR Oculo-dento-osseous syndrome OR Oculo-dento-osseous syndrome OR Oculo-dento-osseous dysplasia OR Oculo-dento-osseous dysplasia OR ODDD OR ODOD).

### Screening process

Endnote 20 reference manager was used for article citations. After removing duplicate references, all the remaining articles were screened by two authors (KDVH & FUMR) according to the predetermined inclusion criteria. Later, data was extracted by KDVH on a calibrated predetermined proforma independently, which was rechecked by FUMR.

### Inclusion criteria

- Dental case reports
- Dentistry-related original articles

### Exclusion criteria

- Animal studies/In vitro studies
- Molecular studies (gene analysis)
- Medical case reports/Original articles
- Abstracts only
- Letter to Editors
- Conference proceedings

### Data extraction

For data extraction customized proforma was designed by the authors to extract the required data from included studies:

1. Study details (title, type of study, authors, journal of publication, year of publication).
2. Study characteristics (specialty field, no. of cases included).
3. Age, gender, Oral manifestations, treatment provided, follow-up time.
4. Diagnostic measure (genetic analysis).

## Results

**Fig. 8:**
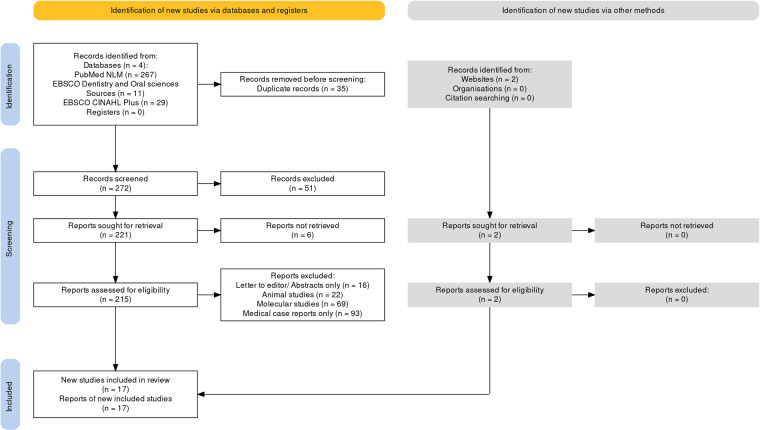
PRISMA flow chart. PRISMA Flow Chart for literature search.

A total of 309 studies were identified after a detailed literature search. After removing duplicates, the number of studies was reduced to 223. After screening these studies by the authors (KDVH & FUMR) following the predetermined inclusion and exclusion criteria, 17 articles were included in this study for final analysis (full text of 6 articles could not be retrieved due to their non-availability in the database). The full screening process is shown in the PRISMA flowchart shown in Fig. 8.

### Characteristics of included studies

The selected articles included dental case reports (*n* = 15), one case report with a review, and one original article. A total of 22 patients (8 = females, 14 = males) are reported with ODDD with dental malformations. Ten out of 17 studies confirmed gene analysis of reported patients. A summary of selected studies is given in Table 2. Below are brief results of the selected studies based on the diagnostic measure, dental manifestations, and their management options.

**Table 2:** Brief review of the selected articles.

Serial no.	Type of study	Journal	Author, year	No. of patients, age, gender	Oral manifestation	Confirmed Genetic analysis	Treatment provided
1	Case report	BMJ Case Reports	Jensen, E. D. [8] (2021)	1 patient 3 years Female	Extensive enamel loss (hypomineralization), enamel fractures in primary teeth	Mutation in one copy of *GJA1* gene (+)	SSCs on primary first molars, sealants on second primary molars
2	Case report and literature review	Annals of Clinical and Laboratory Science	Choi, J. et al. [21] (2018)	1 patient 5 years Male	Primary dentition: multiple dental caries Permanent dentition: Generalized enamel hypoplasia	Missense mutation in *GJA1* (+)	Restorative work like full crowns on primary teeth
3	Case report	Iranian Journal of Child Neurology	Owlia, F. et al. [14] (2017)	1 patient 5 years Male	Maxillary deficiency, premature primary tooth loss	(−)	(−)
4	Case report	International Journal of Prosthodontics	Hadjichristou, C. et al. [22] (2017)	1 patient 25 years Female	Narrow maxilla, microdontia, spaced dentition, curved root morphology, multiple periapical abscesses, pulp stones, distally inclined condyles	Missense mutation in *GJA1* (+)	Definitive metal-ceramic, full-coverage restoration in maxilla and mandible
5	Original article	Oral Diseases	Porntaveetus, T. et al. [23] (2017)	1 patient 2.6 years Male	Severe deterioration of teeth due to hypoplasia	Mutation in *GJA1* (+)	Extraction of maxillary incisors, SSCs on all other erupted teeth with or without pulpectomy
6	Case report	Journal of American Academy of Physician Assistants	Mills, J. K. et al. [15] (2015)	1 patien 9 months Female	Small teeth, white spots on her enamel	(−)	Routine dental checkup to monitor enamel irregularities
7	Case report	Journal of Dental Research	Amano, K. et al. [24] (2012)	1 patient 3 months Male	Bilateral cleft lip and cleft palate	mutation in *GJA1* (+)	Cleft lip and palate surgically repaired
8	Case report	Journal of Oral Science	Aminabadi, N. A. et al. [25] (2010)	1 patient 8 years Male	Hypodontia, small hypoplastic teeth, pulp calcification, lack of contrast between enamel and dentin, taurodontism	Missense mutation in *GJA1* (+)	Extraction of unrestorable teeth, pulpotomy of immature asymptomatic teeth, root canal treatment apexification of immature symptomatic teeth, direct composite veneer on anterior and Stainless-steel crowns (SSCs) on premolars teeth
9	Case report	Journal of Clinical Pediatric Dentistry	Aminabadi, N. A. et al. [26] (2009)	1 patient 8 years Male	Dome-shaped Palate, enlarged mid-palatal raphe, Mamelon-shaped tip of the tongue, smaller hypoplastic teeth, unclear lamina dura, decreased thickness of dentin, lack of contrast between enamel and dentine, taurodontism	Markers from chromosome 6q22-q23 (+)	Under treatment by multidisciplinary dental team
10	Case report	American Journal of Medical Genetics Part- A	Feller, L. et al. [27] (2008)	1 patient 11 year Male	Hypoplastic enamel, dental abscess, taurodontism	Missense mutation in *GJA1* (+)	(−)
11	Case report	International Journal of Oral and Maxillofacial Surgery	van Es, R. et al. [28] (2007)	1 patient 10 years Female	Hypoplastic enamel, retrognathic mandible	Mutation in *GJA1* (+)	Routine dental follow-up
12	Case report	Dentomaxillofacial Radiology	Scheutzel, P. [16] (1991)	1 patient 26 years Male	Generalized hypoplastic enamel, hypocalcified and exposed dentin, loss of vertical dimension, pulp stones, hypoplastic coronoid process, broad mandibular body, and ramus, hypercementosis	(−)	(−)
13	Case report	Oral Surgery Oral Medicine Oral Pathology	Schuller, M. G. et al. [17] (1986)	1 patient 35 years Males	Maxillary hypoplasia, enlarged mandible, severe tooth wear on standing left mandibular canine, premolar and right mandibular second molar teeth	(−)	(−)
14	Case series	Acta Paediatrica Scandinavica	Thodén, C. J. et al. [18] (1977)	4 patients 4 years Female 3 years Female 28 years Male 2 years Male	Enamel defects, poor mineralization	(−)	(−)
15	Case report	Oral Surgery Oral Medicine Oral Pathology	Zach, G. A. [19] (1975).	1 patient 10 years Female	Generalized enamel hypoplasia, pulp stones	(−)	(−)
16	Case series	Oral Surgery Oral Medicine Oral Pathology	Eidelman, E. et al. [20] (1967)	3 patients 10.5 years Male 10 years Male 3 years Female	Cleft lip and palate, missing maxillary right central incisor and mandibular right second premolar, multiple carious teeth enamel hypoplasia	(−)	(−)
17	Case report	Journal of Pediatrics	Gorlin, R. J. et al. [7] (1963)	1 patient 12 year Male	Severely hypoplastic yellow discolored teeth, pulp stones, short mandibular ramus and body, absent frontal sinus	(−)	Extraction and prosthesis

### Diagnostic measures

As previously mentioned, ODDD is a genetic disorder caused by a missense mutation in the GJA1 gene that encodes for a transmembrane protein (Cx43). Eight studies reported diagnosis based on clinical features [7, 14–20], while nine studies confirmed missense mutation in GJA1 at chromosome 6q22-q23 by genetic analysis [8, 21–28].

### Dental manifestations

Enamel hypoplasia, hypomineralization, microdontia, pulp stones, curved roots, taurodontism, discolored teeth, tooth loss with or without caries, and peri-apical abscess are common findings in ODDD patients [7, 8, 15–19, 21–23, 25–28]. Other rare findings, i.e., cleft lip and palate [20, 24] short mandibular ramus and body, absent frontal sinus [7], distally inclined condyles [22], and hypoplastic maxilla [14, 17, 22] may also be present.

### Management and follow-up

Management described below was reported by selected articles and was primarily based on the minimum invasive options:

#### Primary teeth

Extraction of grossly carious teeth, deep fissure sealants, early restoration of dental caries, prevention of tooth wear with stainless steel crowns (SSCs) in posterior teeth with or without pulpectomy was carried out in studies [8, 21, 23].

#### Permanent teeth

Extraction of unrestorable teeth, restoration of caries, pulpotomy in immature asymptomatic teeth, root canal treatment (apexification in immature teeth) in symptomatic teeth followed by full coverage restorations were done in studies [22]. A regular, usually 3-monthly dental evaluation of restored teeth and unrestored dentition is recommended to monitor and early management of any abnormality [8, 22].

## Discussion

Oculo-dento-digital dysplasia is an uncommon condition that is rarely recognized by dentists,

For this reason, we decided to write this report along with a literature search so that the readers can become more familiar with this condition to better serve their patients.

In our review, we found that ODDD is an autosomal dominant genetic disorder, which is characterized by abnormal ocular, dental, and digital findings. It is caused by a mutation in GJA1 gene encoding Cx43 [9, 29]. In our case, the patient presented with multiple dental caries, enamel hypoplasia, pulp stones, and taurodontism and syndactyly, which are typically digital and dental manifestations of this syndrome. Genetic analysis of 178 genes (List given in genetic analysis report) responsible for limb and digital malformations was done for this patient. An uncertain significance of heterozygous variant for DLX6 (Distal-less homeobox) [99_119del (p.Gln38_Gln44del)] which is responsible for autosomal dominant split-hand/foot malformation type 1 [30] and GJA1 [c.196 T > C (p.Tyr66His)] gene involved in autosomal dominant and recessive oculodentodigital dysplasia was identified (9). DLX6 gene abnormality rarely shows dental involvement usually crowding [31]. However, the facial (extra-oral) features were mild but consistent with ODDD (autosomal dominant), showing a thin nose with hypoplastic alae nasi, short palpebral fissures. The present case suggests, ODDD should be considered even when ocular symptoms are un-remarkable and this correlates with the previous literature that there is approximately 70% chance of ocular manifestations in ODDD patients [9].

The other conditions that have similar features to ODDD are amelogenesis imperfecta (AI), oral-facial-digital syndrome, Hallerman-Streiff syndrome (HSS) [32], and Saethre-Chotzen syndrome. ODDD can be differentiated from AI, as the later condition shows little systematic involvement [33]. Oral-facial-digital dysplasia involves the renal system and has features like lobulated tongue without ocular manifestations which differentiates it from ODDD [34]. HSS may share similar clinical ocular and dental features with ODDD but the presence of the skin conditions, dwarfism differentiate it from ODDD [35]. Saethre-chotzen syndrome has features of the characteristic craniosynostosis, ptosis, and absence of any dental manifestations that differentiate it from ODDD [36]. Further details are given in Table 3.

**Table 3:** Differential diagnosis of ODDD.

	ODDD	Amelogenesis Imperfecta	Oro-facial-digital syndrome	Hallermann-Streiff syndrome	Saethre-Chotzen syndrome
Eye Features	Microcornea and microphthalmia	Not-present	Not-present	Cataracts congenitally, Microphthalmia, Ptosis	Ptosis, Strabismus, Blepharospasm
Dental/oral Findings	Hypoplastic enamel and small teeth	Hypoplastic/hypomineralized/hypoplastic and discolored teeth	Lingual hamartoma, Lobulated tongue, Cleft palate	Hypoplastic enamel, compromised teeth	Not-present
Digital Findings	Syndactyly, Clinodactyly	Not-present	Syndactyly, Clinodactyly	Not-present	Syndactyly
Extra-oral Findings	Long narrow and thin nose, Hypoplastic alae nasi, Thin nostrils, Small anteverted nares	Not-present	Hypertelorism, Cleft lip	Dyscephalia, Pinched beaked nose, Retrognathia, Everted lip, High palate	Craniosynostosis, Low frontal hairline, Facial asymmetry, Antihelix abnormality
Other Sytematic Findings	Spastic gait, Hyperreflexi, Conductive hearing loss, Hypotrichosis	Nephrocalcinosis	Renal dysplasia, Polycystic kidney	Proportionate dwarfism, Skin atrophy, Hypotrichosis	Not-present
Genes Affected	GJA1 AMELX ENAM KLK4 LAM3 MMP20	DLX3 WDR72 GPR68 FAM83H FAM20A	OFD1 C5orf42	GJA1	TWIST1 FGFR2

In the present case, the primary goal was to treat the dental disease (i.e., pulpitis and tooth wear) and seal the other teeth for the preservation of arch integrity for patients well being (nutritional, esthetic, and psychological). Teeth were structurally compromised, thus prone to caries and fractures from trauma. Though the symptomatic management in ODDD patients is the same as non-syndromic patients in a few situations, special considerations are required for long-term prognosis, i.e., preservation of teeth by sealing the deep pits and fissures to prevent caries [22]. Conservative treatment plan was of utmost importance as extraction could have led to compromised development of alveolar bone. In ODDD patients, the remodeling process may not be as efficient as in unaffected population due to lack of coordinating events in the alveolar bone due to alteration in Cx43 (29). This can affect the ossteo-integration in case of implant placement or remodeling process in orthodontic movement, thus preservation of alveolar bone is of great importance [22].

## Conclusions

In our patient after genetic analysis, clinical and radiological findings were consistent with ODDD. The primary goals of dental treatment in patients with ODDD should be intended to correct the current oral condition and prevent further tooth loss for maintaining masticatory efficiency, phonetics, and esthetics. In these cases, dentists, pediatric dentists, orthodontists, prosthodontists should work with coordination and multidisciplinary approaches should be provided to improve quality of life.
